# Cardiac assessment of patients during post COVID-19 recovery phase: a prospective observational study

**DOI:** 10.21542/gcsp.2022.18

**Published:** 2022-12-30

**Authors:** Deepak Sharma, Amit Rohila, Surender Deora, Mahendra Kumar Garg, Sanjeev Misra

**Affiliations:** 1 Department of General Medicine, All India Institute of Medical Sciences, Jodhpur, Rajasthan, India; 2 Department of Cardiology, All India Institute of Medical Sciences, Jodhpur, Raj, India; 3 Department of Surgical Oncology, All India Institute of Medical Sciences, Jodhpur, Rajasthan, India

## Abstract

**Introduction:** The coronavirus disease 2019 (COVID-19) is caused by the severe acute respiratory syndrome coronavirus 2 (SARS-CoV-2). It usually presents as a respiratory syndrome but also known to cause many cardiovascular complications during acute phase. However, little is documented about cardiac complications during the post COVID-19 recovery phase. Therefore, this prospective observational study was planned to evaluate cardiovascular effects of the disease in patients recovering from COVID-19.

**Methods:** This was a prospective observational study with a total of 63 patients presenting at 6-month follow-up in post COVID-19 outpatient clinics. Patients with known cases of underlying ischemic heart disease, cardiomyopathy, or any other cardiac disorder, and patients with chronic lung disorder or severe anemia were excluded.

**Results:** Dyspnea was the most common presenting complaint. In biochemical parameters, none of them showed any significant difference between these two groups including NT pro BNP, ferritin, CK-MB. But NT pro BNP was high in moderate/severe cases, especially those having reduced ejection fraction. On echocardiographic evaluation, LVEF was low in moderate/severe group patients (59.7% vs. 51.1%, *p* < 0.0001). LV diastolic dysfunction was also observed more commonly in moderate/severe group patients (55.9% vs. 86.2%, *p* = 0.009). There was no significant difference in RV function assessment parameters.

**Conclusion:** Patients in the moderate/severe group during index hospitalization for COVID-19 should be followed up with NT Pro BNP and echocardiography. This may help in early recognition of heart failure during follow-up of COVID-19 patients.

## Introduction

The coronavirus disease 2019 (COVID-19) caused by the severe acute respiratory syndrome coronavirus 2 (SARS-CoV-2) first appeared in Wuhan, China^1^. Pulmonary involvement in this disease is well known and it usually presents as a respiratory syndrome, commonly with fever and cough. In severe form, it usually presents as acute respiratory distress syndrome and around 5% of patients may require intensive care management.^2^ COVID-19 is also known to cause many cardiovascular complications like acute cardiac injury, arrythmias and various thromboembolic events (VTE)^3^. Most of these events are reported during the active disease process but a little is documented about the cardiovascular effect during the post COVID-19 recovery phase^4,5^. Therefore, this prospective observational study was planned to evaluate cardiovascular effect of the disease in patients recovering from COVID-19.

## Methods

### Study population

This was a prospective observational study conducted at tertiary care center in a western part of India. A total of 63 patients presenting in post COVID-19 outpatient clinic, irrespective of their initial disease severity status, were included. The patients were categorized as mild, moderate and severe as per World Health Organization (WHO) categories for COVID-19 severity. Then for the study purpose, they were divided into two groups as mildly symptomatic (34 patients) and moderate to severe symptomatic (29 patients) during hospitalization.

### Inclusion criteria

1. Patients with age ≥ 18 years
2. History of confirmed COVID-19 by Reverse Transcription Polymerase Chain Reaction (RT-PCR)

### Exclusion criteria

1. Patients with known case of underlying ischemic heart disease, cardiomyopathy, or any other cardiac disorder
2. Patients with chronic lung disorder
3. Patients with severe anemia (hemoglobin <7gm %)

Written informed consent was taken from all study participants. The data was collected with details of clinical signs and symptoms, electrocardiogram (ECG) findings, markers of cardiac injury (Troponin I, CPK-MB), and NT-Pro BNP at 6-month follow -up. Detailed echocardiography was also performed in all patients with focus on biventricular function and strain imaging of right ventricle.

The study was approved by the Institutional Ethics Committee (AIIMS/IEC/2020-21/3070).

### Echocardiographic examination

All patients underwent complete echocardiography examination with commercially available ultrasound system (EPIC 7C, Philips Medical Systems). The following echocardiographic views were recorded: parasternal long-axis views (PLAX), parasternal short-axis views (PSAX) and apical long-axis four-chamber views.

**Left ventricular (LV) function** was assessed by LV volumes, ejection fraction (LVEF), and tissue Doppler imaging. LVEF was assessed by modified biplane Simpson method and <50% was taken as abnormal. LV diastolic function was assessed in standard apical four chamber view by measuring mitral E and A waves, Tissue Doppler Imaging (TDI) e’ and a’ waves. Grade 1 LVDD was defined by an E/A ratio <0.8, and E/e’ (septal) <8 while Grade 2 LVDD was defined as E/A ratio 0.8–1.5, and E/e’ (septal) 9–14. Type 3/4 was defined as E/A >2.

**Right ventricular (RV) function** was assessed by tricuspid annular plane systolic excursion (TAPSE), RV Sa and RV free wall longitudinal strain (FWLS). TAPSE was measured in M-mode at the level of the lateral tricuspid valve annulus and <16 mm was considered as abnormal. RV Sa was measured as highest systolic velocity at the lateral tricuspid valve annulus in tissue Doppler imaging and <10 cm/s was taken as abnormal. RV FWLS was calculated as the mean of the basal, mid, and apical strain values after excluding the septal strain values by using speckle tracking software (QLAB 10; Philips Medical Systems). The normal reference values were taken as −26 ± 3.1 for women and −24 ± 2.6 for men and FWLS greater than −20% was considered abnormal.

### Statistical analysis

Statistical analysis was performed using SPSS version 23 (SPSS, Inc, Chicago, IL). Continuous variables were checked for normality. Normally distributed variables are present”-ed as the mean ± standard deviation and non-normally distributed variables are presented as the median (interquartile range). Categorical data were presented as number (%). Differences between the groups were analyzed using Student’s T-test.

## Results

The mean age of patients having mild symptoms was 47.9 ± 14.8 years and of those having moderate/severe symptoms was 51.1 ± 12.6 years (Table 1). The majority of patients were male in both groups (74% in mildly symptomatic and 83% in moderate to severe symptomatic). There was no significant difference in comorbidities between the two groups.

**Table 1:** Demographic characteristic and clinical profile of study population.

	Population (n =63)	Mild symptomatic (n =34)	Moderately/severe symptomatic (n =29)	P value
**Characteristic**				
Age, in years (mean (SD))	49.4 ± 13.8	47.9 ± 14.8	51.1 ± 12.6	0.36
Male	49 (77.77)	25 (73.52)	24 (82.75)	0.38
**Comorbidities**				
Hypertension, n (%)	18 (28.57)	8 (23.53)	10 (34.48)	0.41
Diabetes mellitus, n (%)	9 (14.28)	4(11.76)	5(17.24)	0.13
**Presenting complaints**				
Dyspnea, n (%)	36 (57.14)	15 (44.11)	21 (72.41)	0.04
Chest pain, n (%)	12 (19.04)	6 (17.64)	6 (20.68)	0.75
Palpitations, n (%)	2 (3.17)	1 (2.94)	1(3.44)	0.09
Fatigue, n (%)	16 (25.39)	10 (29.41)	6 (20.68)	0.42
Heart rate (beats/min)	79	77	80	0.53

Dyspnea was the most common presenting complaint and was present in 36 patients out of 63 (57.1%). The patient group with moderate to severe symptoms presented more commonly with dyspnea as compared to group with mild symptoms (*p* = 0.04). There was no other significant difference between the two groups in presenting symptoms like chest pain, fatigue and palpitations.

In biochemical parameters, there was no significant difference between the groups, including NT pro BNP, ferritin, CK-MB. But NT pro BNP was high in moderate/severe cases, especially those having reduced ejection fraction (Table 2). The average heart rate of the patients in moderate-severe symptom category was 80 per minute and in mild symptomatic category it was 77 per minute and the difference was statistically not significant.

**Table 2:** Biochemical parameters in patients with mild disease versus moderate/severe illness initially.

Biochemical parameters	Population (n =63)	Mild disease (n =34)	Moderate/severe disease (n =29)	P value
NT pro BNP, median (IQR)	49 (IQR 35–98.8)	48(IQR 30–97)	57(IQR (30–106)	0.263
Ferritin (ng/ml)	88.2 ± 88.1	72.6 ± 63.3	95.3 ± 90.8	0.56
CK-MB (ng/ml)	14.4 ± 4.4	14.4 ± 4.6	14.3 ± 4.2	0.99
LDH (IU/L)	200.4 ± 15.6	193.7 ± 52.4	208.1 ± 48.3	0.26

### Echocardiographic evaluation

Left ventricular function assessment was done with measurement of LVEF and LV diastolic function (Table 3). LVEF was found to be significantly low in moderate/severe group patients (59.7% vs. 51.1%, *p* < 0.0001). Four patients in moderate/severe group had LVEF <50% and had longer hospital stay as compared to other patients. Similarly, left ventricular diastolic dysfunction (LVDD) was observed more commonly in moderate/ severe group patients (55.9% vs. 86.2%, *p* = 0.009).

**Table 3:** Echocardiographic characteristics at 6-months post COVID-19 in patients with mild disease versus moderate/severe illness initially.

Echo parameters	Population (n =63)	Mild disease (n =34)	Moderate/severe disease (n =29)	P value
**LV assessment**				
LVIDd (mm)	46.0 ± 6.0	54 ± 5.7	48 ± 5.3	0.62
LVIDs (mm)	33.0 ± 7.0	30 ± 5.6	36 ± 7	0.83
LVEF (%)	55.8 ± 9.7	59.7 (3.9)	51.1(12.2)	<0.0001
E/A ratio	0.98 ± 0.36	1.06 ± 0.37	0.9 ± 0.34	0.65
Septal e’ (mm/sec)	8.9 ± 2.4	9.28 ± 1.77	8.6 ± 2.9	0.04
E/e’	8.1 ± 2.5	8.13 ± 1.4	8.11 ± 3.35	0.97
LVDD, n (%)	48 (76.2)	19 (55.9)	29 (86.2)	0.009
**RV assessment**				
RVSP (mm Hg)	30.58 ± 7.3	30.4 ± 6.4	30.9 ± 8.4	0.78
TAPSE (mm)	2.3 ± 1.9	2.09 ± 0.29	2.02 ± 0.19	0.28
RV s’ wave (mm/s)	12.5 ± 1.7	12.5 ± 1.7	12.4 ± 1.8	0.86
PAT (msec)	117.5 ± 12.1	117.4 ± 11.2	117.7 ± 13.2	0.93
RV strain basal (%)	−28.45 ± 7.3	−28.5 ± 6.8	−28.4 ± 7.8	0.99
RV strain mid (%)	−24.07 ± 6.6	−27.3 ± 6.4	−21.1 ± 5.2	0.002
RV strain apex (%)	−24.2 ± 7.5	−23.9 ± 8.8	−24.5 ± 6.3	0.8
RV FWLS (%)	−26.9 ± 8.2	−28.58 ± 8.4	−25.5 ± 7.5	0.14

There was no significant difference in RV function assessment parameters like TAPSE, RV s’ and RV FWLS. RV strain imaging in mid segment was significantly higher in moderate/severe group patient (−27.3 ± 6.4 vs. −21.1 ± 5.2, *p* = 0.002) whereas there was no significant difference in strain imaging at RV apex and base.

## Discussion

COVID-19 is primarily a respiratory disease with some recent studies suggesting a direct cardiomyocyte tropism for SARS-CoV 2. As a consequence of this, there can be myocarditis, development of left ventricular systolic and diastolic dysfunction, and myocardial fibrosis^4,5^. There can also be RV dysfunction in these patients because of predominant and significant pulmonary involvement.

Different studies have reported myocardial injury during the acute COVID-19 phase, but echocardiographic follow-up studies are limited. Therefore, this study was planned to assess LV and RV function by 2D echocardiography during 6-month follow-up in patients who had suffered COVID-19 and were hospitalized.

Our findings revealed a significant diastolic dysfunction of left ventricle in those patients who had moderate/severe COVID-19 initially. LVDD was found in 48 patients out of 63 (76%), 12 had grade 2 LVDD, and 36 had grade 1 LVDD suggesting patients with moderate/severe COVID-19 are more likely to develop LVDD at 6- months follow up. Our study also revealed a significantly low LVEF in those patients who had suffered moderate/severe disease initially as compared to those with milder illness. Four patients out of 63 (6.3%) were found to have significant left ventricular systolic dysfunction (<50%), which was comparable to the data in previous study which showed 8.8% left ventricular systolic function^6^. These four patients also had longer hospital stay as compared to other patients with LVEF >50% and higher NT pro BNP level at follow up. Therefore, patients with longer hospital stay should be followed with echocardiographic evaluation and markers of cardiac injury during follow-up.

RV function assessment is also important in follow-up of these patients specially those having persistent dyspnea. Right ventricular longitudinal strain pattern can assess intrinsic myocardial performance and can differentiate active movement from passive movement. Our study did not reveal any significant difference in RV function between the two groups except the RV mid wall strain indicating abnormally decreased shortening of this region during systole. Other right ventricular parameters like RVSP, TAPSE, pulmonary acceleration time and s’ velocity were not significantly different.

NT-pro BNP is a quantitative plasma biomarker usually reflecting hemodynamic cardiac stress and, therefore, play a central role in the diagnosis and management of heart failure (HF)^7^. It was noted that NT-pro BNP levels were high in moderate/severe cases but not statistically significant. High NT pro BNP level also correlated with the presence of dyspnea as chief complain. Most of the previous studies demonstrated higher levels of NT pro BNP during acute COVID 19^8^, but to our knowledge none of them demonstrated any persistently high levels during follow up, as in our study. CK-MB and ferritin levels as marker of cardiac myocyte injury and inflammation, respectively, found to have no significant difference between these two groups.

COVID-19 predominantly affects lung parenchyma, and thus the involvement of right side of heart was expected more, but contrary to that, in our study, left ventricular dysfunction was found more significant in patients with initial moderate/severe COVID-19. It can be explained by the direct cardiac myocyte injury by SARS-CoV-2 as discussed earlier. Longer follow-up and large studies will be needed to understand if this pattern of cardiac ventricular dysfunction reflects a chronic pathological remodeling or a progressive recovery after the initial myocyte injury to the heart.

## Conclusion

Echocardiographic evaluation in patients with moderate/severe COVID-19 infection and persistent dyspnea helps in clinical assessment during follow-up. LV dysfunction was present significantly higher in moderate/severe group patients as compared to mild cases without any significant difference in RV function. In addition to this, assessment of NT-pro BNP during follow-up period helps in monitoring of heart failure in these patients.
